# Supplementary material for: Tead1a Initiates Transcriptional Priming Through the TEAD1a/YAP‐Notch1‐Spi1/Cebpα Axis to Promote Neutrophil Fate
Source: Adv Sci (Weinh). 2025 Aug 28;12(41):e05441. doi: 10.1002/advs.202505441 (PMC12591127; doi:10.1002/advs.202505441)
Supplement: Supplementary file 4 — Supplementary Dataset [file ADVS-12-e05441-s004.zip › 3-4.zip!/3-4/02-4.cluster/cluster11/Enrich/ego_kegg.pdf]

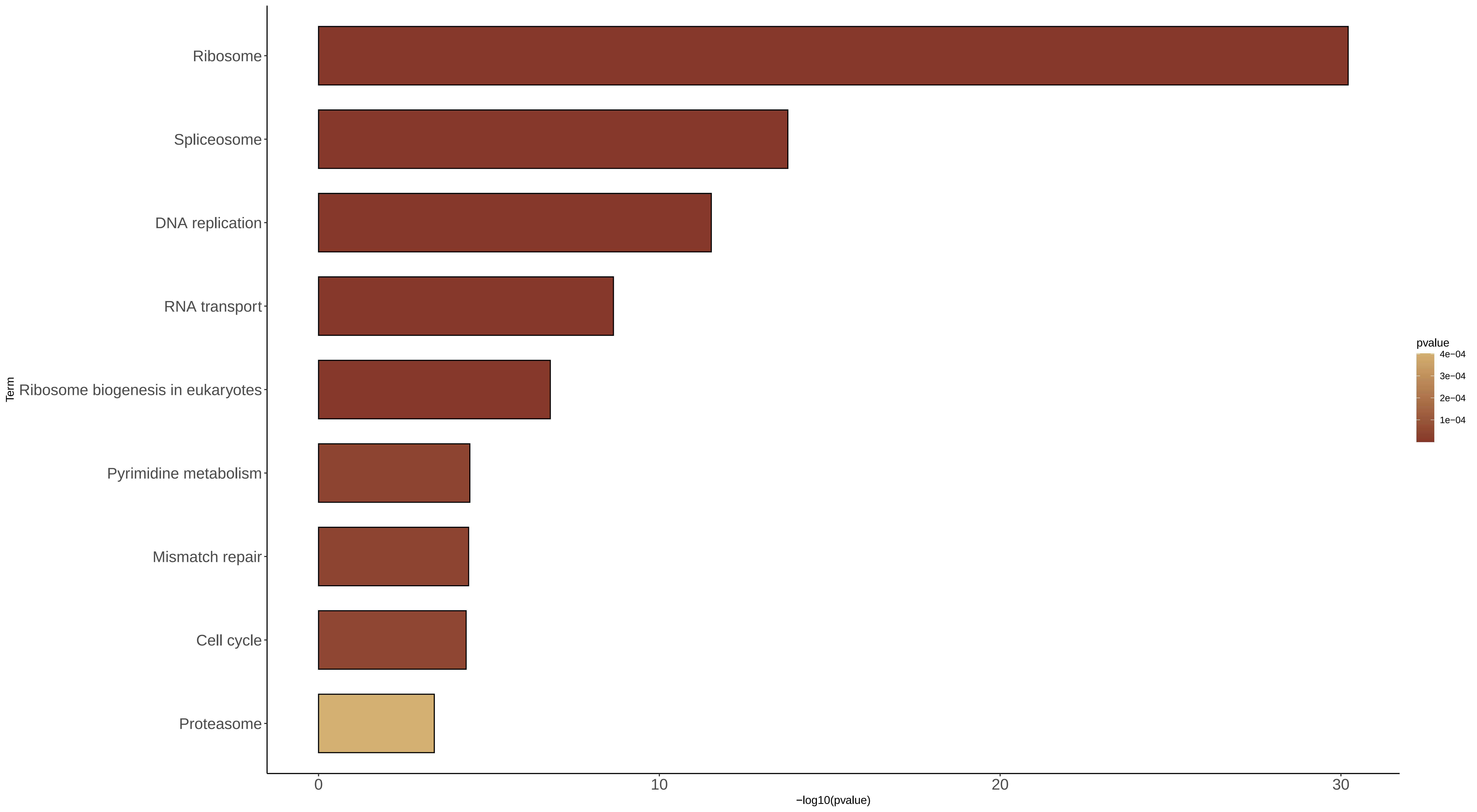
Ribosome
Spliceosome
DNA replication
RNA transport
Ribosome biogenesis in eukaryotes
Pyrimidine metabolism
Mismatch repair
Cell cycle
Proteasome
Term
0
10
20
30
−log10(pvalue)
pvalue
4e−04
3e−04
2e−04
1e−04
